# A mouse-to-man candidate gene study identifies association of chronic otitis media with the loci *TGIF1* and *FBXO11*

**DOI:** 10.1038/s41598-017-12784-8

**Published:** 2017-10-02

**Authors:** Mahmood F. Bhutta, Jane Lambie, Lindsey Hobson, Anuj Goel, Lena Hafrén, Elisabet Einarsdottir, Petri S. Mattila, Martin Farrall, Steve Brown, Martin J. Burton

**Affiliations:** 1 Brighton and Sussex University Hospitals NHS Trust, Eastern Road, Brighton, BN2 5BE UK; 2 Nuffield Department of Surgical Sciences, University of Oxford, Headley Way, Oxford, OX3 9DU UK; 3 MRC Harwell Institute, Harwell, OX11 0RD UK; 4 Division of Cardiovascular Medicine, Radcliffe Department of Medicine, University of Oxford, Oxford, OX3 9DU UK; 5 Wellcome Trust Centre for Human Genetics, University of Oxford, Oxford, OX3 7BN UK; 6 Department of Otorhinolaryngology, Helsinki University Central Hospital, Helsinki, HUS Finland; 7 Folkhälsan Institute of Genetics, and Molecular Neurology Research Program, University of Helsinki, Helsinki, Finland; 8 Department of Biosciences and Nutrition, Karolinska Institutet, Huddinge, Sweden; 9 The UK Cochrane Centre, Summertown Pavilion, 18–24 Middle Way Oxford, Oxford, OX2 7LG UK

## Abstract

Chronic otitis media with effusion (COME) is the most common cause of hearing loss in children, and known to have high heritability. Mutant mouse models have identified *Fbxo11*, *Evi1*, *Tgif1*, and *Nisch* as potential risk loci. We recruited children aged 10 and under undergoing surgical treatment for COME from 35 hospitals in the UK, and their nuclear family. We performed association testing with the loci *FBXO11*, *EVI1*, *TGIF1* and *NISCH* and sought to replicate significant results in a case-control cohort from Finland. We tested 1296 families (3828 individuals), and found strength of association with the T allele at rs881835 (p = 0.006, OR 1.39) and the G allele at rs1962914 (p = 0.007, OR 1.58) at *TGIF1*, and the A allele at rs10490302 (p = 0.016, OR 1.17) and the G allele at rs2537742 (p = 0.038, OR 1.16) at *FBXO11*. Results were not replicated. This study supports smaller studies that have also suggested association of otitis media with polymorphism at *FBX011*, but this is the first study to report association with the locus *TGIF1*. Both *FBX011* and *TGIF1* are involved in TGF-β signalling, suggesting this pathway may be important in the transition from acute to chronic middle ear inflammation, and a potential molecular target.

## Introduction

Chronic inflammation of the middle ear (otitis media, OM) causes persistent and sometimes permanent disability, due to middle ear effusion or irreparable damage to middle ear tissues. Chronic otitis media with effusion (COME) is the prototypical form of chronic middle ear inflammation in high-income countries, where the middle ear becomes filled with a sero-mucoid effusion (“glue ear”) secreted by inflamed mucosa, which interferes with the conduction of air-borne sound. In the USA, COME has an estimated annual incidence of 6–7% in two year old children^1^, making it the most common cause of childhood hearing loss, and the insertion of ventilation tubes (“grommets”) to treat COME the most common operation in children. Children and adults with chronic otitis media report lower disease-specific and general quality of life^2,3^, and persistent hearing loss may cause detriment to linguistic development and education^3^. In the USA the total healthcare expenditure attributable to otitis media in children exceeds $5 billion per year^4^.

Although the sequence of events leading to acute OM (AOM) is fairly well understood, the reasons for chronic OM are less so. In AOM, bacteria resident in the nasopharynx ascend the Eustachian tube to enter the middle ear space, typically leading to a suppurative effusion, accompanied by symptoms of pain and fever^1^. The vast majority of cases of AOM resolve without significant sequelae, but in a minority inflammation does not resolve, leading to the persistent non-suppurative effusion that characterises COME. COME can also develop without prior history of AOM, through mechanisms that are unclear^1^.

The reasons and biology underlying the transition from acute to chronic OM are not known, but epidemiological studies suggest host genetics play a critical role. In a study of twins, Casselbrant reported that time with middle ear effusion in COME had heritability of 71%^5^. Others have shown that treatment of OM with grommets is associated with a five fold risk of the same treatment in first degree relatives^6^.

Interrogating the genetic architecture of OM risk can be achieved through genome-wide or candidate gene association studies. All disease cohorts for OM reported thus far are under-powered for performing Genome-wide association studies (GWAS). GWAS also carries a risk that critical functional variants in candidate genes might not be included on SNP arrays, or cannot be reliably imputed.

The candidate gene approach overcomes the difficulties of GWAS, but ascertaining relevant loci for testing can be difficult. Genetically altered animal models are one means of interrogation, and in OM research the mouse has become the preferred animal model, not least because of the toolkit available to genetically alter this species^7^. A recent review^8^ identified three non-syndromic mouse models of chronic OM: the *Jeff* mouse^9^, *Junbo* mouse^10^, and *Tgif1* knockout mouse^11^. All three of these mice have been identified by members of our research group. Since then we have reported one additional non-syndromic mouse model of chronic OM: the *Edison* mouse^12^ (Table 1). The loci mutated in these models are respectively *Fbxo11*, *Evi1* (part of the *Mecom* cluster), *Tgif1*, and *Nisch*. These represent good loci for evaluating candidate genes underlying susceptibility to COME in man.

**Table 1:** Mouse models of chronic otitis media discovered at MRC Harwell. All develop spontaneous chronic OM within a few weeks of birth.

Name	Locus	Background	Discovery mechanism	Reference
*Jeff* (het)	*Fbxo11*	C3H/HeN	ENU mutagenesis	Hardisty-hughes *et al*.^9^
*Junbo* (het)	*Evi1 (Mecom)*	C3H/HeN	ENU mutagenesis	Parkinson *et al*.^10^
*Tgif1 knockout*	*Tgif1*	C57BL/6J	Knockout	Tateossian *et al*.^28^
*Edison* (hom)	*Nisch*	C3H/HeH	ENU mutagenesis	Crompton^12^

Most existing association studies in the field of OM genetics have been poorly phenotyped and used small sample sizes^13^, which carries significant penalty to statistical power. Here we report the largest gene association study to date for OM, where we focus on a cohort of well-phenotyped children with COME, and we test for association with the four candidate loci identified through mouse models. Using the transmission disequilibrium test (TDT), we report positive association with the loci *TGIF1* and *FBXO11*, but no evidence of association with the loci *EVI1* or *NISCH*.

## Results

### Ethical Approval

The UK National Research Ethics Service granted national approval for this study (reference 08/H0605/109), and the study was conducted in accordance with their guidelines and with the study protocol. All participants gave informed consent to participation in the study.

### Cohort Recruitment

The cohort comprised 1296 children undergoing treatment for COME (as defined in Methods), and their parents, giving a total sample of 3828 individuals. This sample size provides 80% power to detect a common allele (risk allele frequency >0.2) conferring a genotype relative risk of 1.2 controlling the type 1 error <0.05^14^. Of the probands, 57% were male, and 95% described their ethnicity as white. There was a Gaussian distribution of the ages of children at recruitment, with 41% of children aged 4 or 5. All but 2 parents and siblings disease affection status was recorded as “unknown”.

### Tagging Algorithm and Data Cleaning

A total of 53 SNPs were derived for analysis from the tagging algorithm (Table 2).

**Table 2:** Tagging algorithm. SNPs that were force included for *FBXO11* were rs2134056, reported by Segade *et al*.^21^ and rs330787 by Rye *et al*.^20^.

Locus	transcripts	Tagging algorithm configuration					Tagging results		
		Region(NCBI 36 coordinates)	MAF	Population	r^2^	SNPs force included	Number of tag SNPs	Alleles captured	Mean max r^2^
EVI1*	5	3:170282244..170349787	>0.05	CEU	>0.8	—	10	71/71	0.98
*FBXO11*	11	2:47882565..47991318	>0.05	CEU	>0.8	rs2134056, rs330787	17	44/44	0.96
*NISCH*	14	3:52452118..52496071	>0.05	CEU	>0.8	—	2	2/2	1
*TGIF1*	22	18:3399072..3451404	>0.05	CEU	>0.8	—	24	41/41	0.979

^*^EVI1 is part of the MDS1/EVI1 cluster, renamed as MECOM by HUGO.

We processed genotyping data using PLINK (see Methods). Genotyping success rate for all SNPs was >88% meaning that no SNPs were excluded from analysis. No SNPs had MAF <1%. One SNP deviated significantly from HWE and so was excluded. 17 probands and 12 siblings showed excessive evidence of non-Mendelian inheritance; 607 participants (13.3%) had DNA with genotyping success rate <90%. These individuals were excluded from further analysis.

### Association Testing

The top hits (lowest p values) from association testing are shown in Table 3, and all other results in Supplementary Table S1. At the p < 0.05 value, there were four SNPs nominally associated with disease susceptibility. These are the T allele at rs881835 and the G allele at rs1962914 at the *TGIF1* locus, and the A allele at rs10490302 and the G allele at rs2537742 at the *FBXO11* locus.

**Table 3:** Results of TDT association testing for the SNPs evaluated in the UK cohort. Only the top four hits are shown, data for all SNPs tested are shown in Supplementary Table S1. MAF = Minor Allele Frequency.

Locus	SNP	Genomic coordinate and annotation	p-value	Risk allele	MAF	Odds ratio(95% confidence interval)
*TGIF1*	rs881835	18:3449312 (intronic)	0.006	T	0.0711	1.388 (1.099–1.769)
*TGIF1*	rs1962914	18:3426900 (intronic)	0.007	G	0.0290	1.577 (1.140–2.240)
*FBXO11*	rs10490302	2:47812605 (intronic)	0.016	A	0.3129	1.173 (1.030–1.338)
*FBXO11*	rs2537742	2:47811905 (intronic)	0.038	G	0.2535	1.156 (1.009–1.328)

There was no evidence of disease association in this cohort with the two SNPs from the *FBXO11* locus that were force included in the tagging algorithm: rs2130456 (p = 0.276) and rs330787 (p = 0.255). There was also no evidence of association at the *EVI1* or *NISCH* loci.

When the top two hits from the *TGIF1* locus (rs881835 and rs1962914) were analysed as a haplotype block this improved the strength of association to p = 0.0013. There was no increased strength of association when the top two hits from *FBXO11* (rs10490302 and rs2537742) were analysed as a haplotype block (p = 0.086).

### Replication

We undertook replication studies of the top two hits from the discovery cohort in a case-control Finnish cohort (see Methods). We found no strength of association in the replication cohort (both SNPs at p > 0.4).

## Discussion

We have undertaken the largest association study to date for chronic otitis media, and discovered nominal association with the loci *TGIF1* and *FBXO11*. Our discovery cohort has a well-defined phenotype, with analysis only of those with symptoms or signs of COME present for at least 3 months, and effusion confirmed intra-operatively.

Interestingly, many previous studies that have tried to elucidate genetic risk of chronic otitis media have chosen candidate loci involved in innate or acute inflammatory signalling, and have used cohorts that include cases of both recurrent acute and chronic otitis media. Yet several lines of evidence suggest that chronic otitis media should be evaluated and analysed as distinct from acute otitis media. For example, epidemiological studies show that whereas AOM is a risk factor for middle ear effusion, many children with COME have no preceding history of AOM^1^. Transcript analyses in the mouse show that gene expression in otitis media varies with time: genes upregulated in the initial phase of inflammation are different to those found as inflammation resolves^15^, providing evidence of a molecular transition from acute to chronic inflammation. Studies of inflammatory disorders affecting organs other than the middle ear suggest that genetic risk for acute versus chronic inflammation are distinct: homozygous polymorphisms in acute inflammatory response genes rarely cause a significant phenotype, whereas polymorphisms in central regulators of inflammation can lead to chronic inflammation^16^.

Here we have undertaken an analysis specifically of chronic otitis media. Our cohort included only children with COME, and our candidate genes for association testing were those known to predispose to chronic middle ear inflammation in genetically altered mouse models. We found reasonable evidence of association with two SNPs at the *TGIF1* locus (rs881835 and rs1962914) with odds ratios for disease of 1.4–1.6. There was a weaker association with two SNPs within FBXO11 (rs10490302 and rs2537742), with odds ratios of around 1.2. All these SNPs are located in intronic regions, and none of these markers or their proxies are known to cause deleterious protein coding changes (we evaluated SNPs in LD r^2^ > 0.8 on the GRCh37 build of the Ensembl database).

In genetic association studies there is a risk of false discovery, which can be mitigated by replication in an independent cohort. Here we were unable to replicate our findings in the Finnish cohort, reflecting experience from other contexts that many association studies do not in fact replicate^17^. In some cases non-replication may be due to variation in study design, particularly if phenotypes are poorly matched. The Finnish cohort was selected because it used a similar disease definition, but there are some subtle differences: COME was diagnosed as effusion present for 2 months rather than 3 months, and without operative confirmation of effusion. In addition the control group for the Finnish replication was not phenotype clean, which will reduce statistical power. Other reasons for non-replication may be that the genetic structure of the Finnish population is disparate to that of the rest of Western Europe^18^, or that different pathogenic mechanisms exist in different populations. The UK and Finnish cohort have been analysed in a previous study evaluating risk of OM with polymorphism at the *TLR4* locus (with Finnish cohort for discovery and UK cohort for replication), and there again there was failure of replication^19^.

Looking at previous research, two small association studies have reported evidence of association at *FBXO11*. Rye *et al*. showed association with the major A allele at SNP rs330787 in a study from Western Australia of 434 families predominantly suffering from recurrent AOM^20^ (p = 0.009), with replication of this finding in an independent cohort (p = 6.9 × 10–6, OR = 1.55). Segade *et al*.^21^ also reported nominal association at *FBXO11* to the SNP rs2134056 (p = 0.017) in their cohort of 142 families from the US (with a mixed OM phenotype). Whereas we have also found association at the *FBXO11* locus, this is not at the same polymorphisms reported in these other studies. Regardless, these studies, and ours, provide strong evidence that *FBXO11* is associated with risk of otitis media.

The *TGIF1* locus, which was associated with risk of COME, has not been evaluated in any previous genetic association study. The other two loci evaluated in this study, *EVI1* and *NISCH* were not associated with disease. *EVI1* was also not associated with disease in a previous association study for otitis media^22^. To date several small GWAS studies for OM have been reported^23–27^, with no hits near the *TGIF1* or *FBXO11* loci, although these GWAS studies were under-powered to detect alleles of mild or moderate disease risk.

The top hits in this study were at the loci *TGIF1* and *FBXO11*. Both of these loci are thought to regulate TGF-β signalling in the middle ear.

Members of our group have previously evaluated the *Tgif1* knockout mouse^28^, and found that it spontaneously develops chronic bilateral OM within weeks of birth, associated with downregulation of the main executor of Tgf-β signalling, the Smad proteins. In addition our group generated the mutant mouse *Jeff* ^9^, which carries an A1472T transversion in *Fbxo11*. Molecular studies suggest that this mutation disrupts Fbxo11 interaction with the protein p53, with downstream effects increasing levels of Smad2^29^.

Our data therefore suggest that regulation of TGF-β signalling may be critical for the development of persistent inflammation in the middle ear. TGF-β is already implicated as a key regulator of inflammatory response in a number of other organs, through its effects on chemotaxis, activation, and survival of lymphocytes, natural killer cells, dendritic cells, macrophages, mast cells, and granulocytes^30^. Studies in children^31^ and in adults^32^ with COME show that TGF-β1 is consistently found in middle ear effusions, and that levels of this protein correlate to the duration of effusion. SMAD proteins, the downstream effectors of TGF-β signalling, bind to the promoter site of *Muc5ac*, which codes the major mucin found in human middle ear effusion, and disruption of SMAD4 binding significantly impairs induced expression of *Muc5ac* ^33^.

Our previous work has also demonstrated in the *Tgif1* and *Jeff* mouse models (as well as in the *Junbo* model) the presence of hypoxia, and hypoxia responsive molecules such as VEGF^11,34^. Hypoxia may be a common finding in the chronically inflamed middle ear no matter what the aetiology or pathogenesis of disease. However our findings could also reflect cross-talk between Tgf-β and hypoxia signalling pathways^11^, and there is also evidence that FBXO11 directly suppresses HIF-1α^35^ (a key regulator of the hypoxia response in a range of tissue types). In mutant mouse models, VEGF receptor inhibitors have been shown to ameliorate inflammation, and thus represent a potential new therapy^45^. Antagonising TGF-β signalling could (in theory) represent another strategy. However, a key step in realising ambitions for molecular targeting of chronic OM will be the development of methods for local rather than systemic drug delivery.

In conclusion we have performed a genetic association study on the largest cohort to date of children with otitis media. We looked specifically at a well-phenotyped cohort with COME, and tested SNPs at candidate loci derived from genetically altered mouse models of disease. We found evidence of association with the loci *TGIF1* and *FBXO11*, although these findings were not replicated in an independent cohort. Both the *TGIF1* and *FBXO11* loci are thought to be involved in TGF-β signalling, which implies that TGF-β may be critical in the transition from acute to chronic middle ear inflammation, and could be a future target for molecular therapies.

## Materials and Methods

### Recruitment

**Figure 1:**
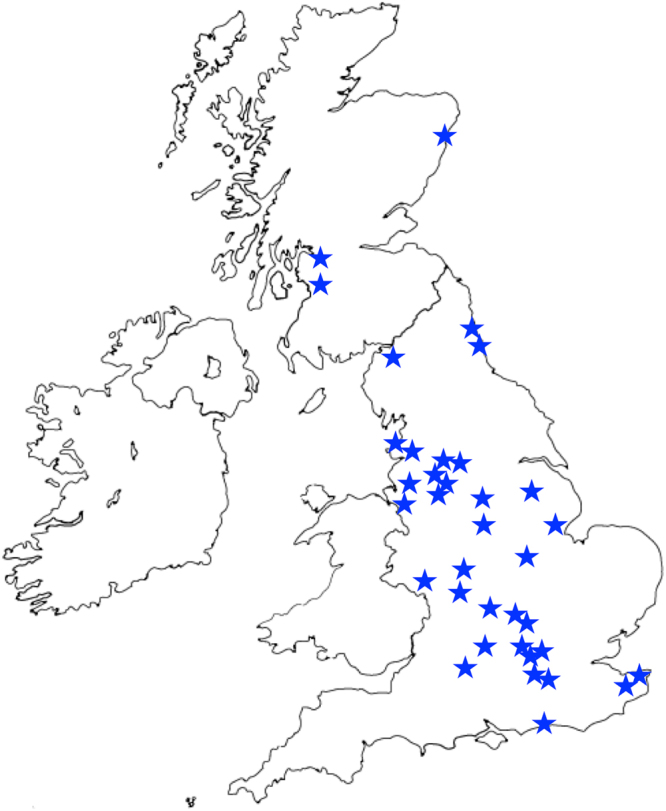
Map of the UK showing recruitment locations for this study, namely hospitals at: Heatherwood, Warwick, Stoke Mandeville, Swindon, Glasgow, Epsom, Northampton, Brighton, Kettering, Derby, Kent, Fairfield, Liverpool. Huddersfield, Chester, Boston, Kings Mill, Manchester, Sunderland, Lincoln, Bradford, Preston, Kidderminister, Worcester, Aberdeen, Kilmarnock, Blackpool, Cumberland, Newcastle, Guildford, Coventry, Milton Keynes, High Wycombe, Oxford, and Wexham Park. Outline map created using InDesign CS6 (8.0) www.adobe.com/products/indesign.html and modified with Powerpoint 2011 products.office.com/en-us/powerpoint.

We recruited children aged 10 and under who were undergoing insertion of grommets (ventilation tubes) from 35 hospitals in England and Scotland (Fig. 1) as a discovery cohort. Children undergoing grommet insertion may have this operation because they have recurrent AOM or COME, and although we recruited children with either of these phenotypes, for association testing we only used the cohort with COME. Here COME was defined as symptomatic effusion lasting at least three months (duration ascertained either from parental report of duration of hearing loss, or serial tympanometry), and effusion confirmed at operation. We excluded children who did not have effusion at operation, and children who had a syndrome leading to craniofacial malformation, immune dysregulation, or anything else that could theoretically alter susceptibility to OM. We also recruited the nuclear family of the proband, to include (where possible) biologically related parents and siblings. The recruitment process is described in more detail elsewhere^36^. Disease status of parents and siblings of the proband was classified as “affected” if they had grommet insertion in childhood, but otherwise as “unknown” (acknowledging that changes in recognition and treatment of OM may vary over time).

### Sample Collection

Saliva was collected from all those recruited using an Oragene® OG-250 pot, aided with saliva sponges for infants (DNA Genotek Inc, Ontario, Canada). Saliva vials were returned by post. DNA was extracted from Oragene® vials using an automated system (LGC, Hoddesdon, UK) where cells were lysed and DNA bound to silica particles mediated by guanidinium isothiocyanate. The solution was washed to remove contaminants and DNA eluted into a low salt buffer. DNA quantification was by spectrophotometry.

### Tagging Algorithm

For each locus we interrogated the Ensembl genome browser (on the GRCh37.p13 assembly) to assess overall single nucleotide polymorphism (SNP). Tagging SNPs were derived using Haploview 4.2 software^37^ from linkage disequilibrium (LD) maps in Phase 3 data of the HapMap (release 28) for the loci *FBXO11*, *EVI1*, *NISCH*, and *TGIF1*. Tracks were configured to include the protein coding region and 3 kb of the untranslated region 3′ and 5′ of this, because no hypothesized regulatory elements (based upon the Ensembl algorithm) were found more distant than this. For the algorithm r^2^ was set to 0.8, minor allele frequency (MAF) to >0.05 in the CEU population, and SNPs associated or approaching significance in previously published studies were force included (Table 2)^20,21^.

### Genotyping

We extracted SNP sequences from the dbSNP database. Primer design and genotyping was performed using the *KASPar* primer extension sequencing, with genotype calling based upon the *Kluster Caller* automated software reading of fluorescence (LGC, Hoddesdon, UK).

Genotyping data were processed using PLINK v1.07^38^. Our data quality control excluded individuals with a genotyping success rate <90%, and SNPs with a genotyping success rate <80%. We tested remaining SNPs for departure from Hardy-Weinberg Equilibrium (HWE), with p < 0.0001 as the criterion for exclusion. Any minor alleles with a frequency of less than 1% in our sample were also excluded.

Remaining data were imported into PedCheck v1.0^39^ and analysed for level 0–2 errors that flag SNPs showing inconsistencies with Mendelian inheritance. If one or two SNPs were discrepant within a nuclear family, discordant genotypes in the parent or sibling were reassigned as unknown. If three or more SNPs were discordant, all data for the discordant individual were excluded from subsequent analyses, on the assumption that this is evidence of high levels of genotype miscall in this individual, or evidence of a non-biological relationship.

Association testing for the loci *FBXO11*, *EVI1*, *NISCH*, and *TGIF1* was performed using TRANSMIT v2.5.4 that extends the Transmission Disequilibrium Test (TDT) to allow for incomplete genotype data and multiple siblings^40^. Any individual who had grommet surgery in the past was classified as affected; all others’ affection status was coded as unknown. Significance of association was assumed at the p < 0.05 level, and odds ratios and 95% confidence limits were also calculated^41^. Where there were several hits at a locus these were re-analysed in haplotype blocks to identify specific risk haplotypes.

### Replication

Significant results from our analysis were submitted for replication in a separate case-control cohort of 402 Finnish children who underwent grommet insertion for recurrent COME (defined as effusion lasting longer than 2 months) and 777 controls (comprising adult Finnish males with no clinical information). Characteristics of this cohort have previously been detailed^19^.

## Electronic supplementary material

Supplementary table S1
