# Supplementary material for: A mouse-to-man candidate gene study identifies association of chronic otitis media with the loci TGIF1 and FBXO11
Source: Sci Rep. 2017 Oct 2;7:12496. doi: 10.1038/s41598-017-12784-8 (PMC5624881; doi:10.1038/s41598-017-12784-8)
Supplement: Supplementary file 1 — Supplementary table S1 [file 41598_2017_12784_MOESM1_ESM.pdf]

A mouse-to-man candidate gene study identifies association of chronic otitis media with the loci *TGIF1* and *FBXO11*

Mahmood F Bhutta
Jane Lambie
Lindsey Hobson
Anuj Goel
Lena Hafrén
Elisabet Einarsdottir
Petri S Mattila
Martin Farrall
Steve DM Brown
Martin J Burton

Supplementary table S1

Results of TDT association testing for all the SNPs evaluated in this study. MAF = Minor Allele Frequency. CI = Confidence Interval

Locus	SNP	p-value	MAF	Odds ratio (OR)	Lower 95% CI for OR	Upper 95% CI for OR
TGIF1	rs881835	0.006433455	0.07108	1.388	1.099	1.769
TGIF1	rs1962914	0.007044058	0.02898	1.577	1.140	2.240
FBXO11	rs10490302	0.016761271	0.3129	1.173	1.030	1.338
FBXO11	rs2537742	0.03786447	0.2535	1.156	1.009	1.328
FBXO11	rs3732191	0.053555504	0.08069	1.234	0.998	1.533
EVI1	rs11721038	0.062347928	0.07554	1.236	0.990	1.551
TGIF1	rs151472	0.063439583	0.2958	1.130	0.993	1.286
FBXO11	rs17395881	0.086931337	0.0978	1.186	0.976	1.446
FBXO11	rs2047681	0.123948822	0.1834	1.131	0.967	1.326
TGIF1	rs238541	0.132462956	0.02653	1.305	0.926	1.870
NISCH	rs1011063	0.133390116	0.1866	1.121	0.966	1.303
NISCH	rs2159607	0.176802287	0.1733	1.112	0.953	1.299
FBXO11	rs960106	0.192890657	0.4002	1.084	0.960	1.225
FBXO11	rs330787	0.255110209	0.3831	1.072	0.951	1.208
FBXO11	rs2134056	0.275733351	0.1697	1.091	0.933	1.278
FBXO11	rs12712997	0.311265125	0.4015	1.063	0.944	1.198
EVI1	rs7633965	0.328022241	0.07036	1.119	0.893	1.407
FBXO11	rs874869	0.333019606	0.4554	1.062	0.940	1.201
EVI1	rs7615880	0.364776995	0.1112	1.088	0.907	1.306
FBXO11	rs2881863	0.387141919	0.06866	1.107	0.879	1.398
TGIF1	rs8082866	0.388810115	0.2416	1.060	0.928	1.212
TGIF1	rs1020301	0.388960245	0.1188	1.083	0.904	1.299
EVI1	rs13090810	0.468018684	0.2065	1.055	0.913	1.219
TGIF1	rs12954964	0.471738859	0.1672	1.059	0.907	1.237
FBXO11	rs13035558	0.473558748	0.2489	1.051	0.917	1.205
FBXO11	rs17395820	0.5021593	0.1041	1.070	0.878	1.305
TGIF1	rs238134	0.506356027	0.2748	1.047	0.914	1.202
EVI1	rs6804720	0.524339148	0.3936	1.040	0.921	1.174
FBXO11	rs7562048	0.555628458	0.4934	1.036	0.920	1.167
TGIF1	rs7229123	0.579866821	0.4337	1.033	0.920	1.162
TGIF1	rs2020436	0.609804305	0.2624	1.036	0.904	1.187
EVI1	rs10936575	0.619152872	0.3838	1.030	0.916	1.160
TGIF1	rs7234567	0.64284546	0.2345	1.033	0.900	1.186
FBXO11	rs2072447	0.671210008	0.2515	1.031	0.897	1.185
TGIF1	rs8082964	0.699521613	0.0967	1.038	0.858	1.258
TGIF1	rs8095532	0.710397742	0.06781	1.047	0.823	1.333
TGIF1	rs7229546	0.713282876	0.2475	1.025	0.898	1.171
EVI1	rs16853239	0.715464448	0.0768	1.043	0.832	1.308
TGIF1	rs11664784	0.720493869	0.2872	1.024	0.899	1.167
EVI1	rs11718015	0.725439848	0.282	1.024	0.898	1.167
EVI1	rs12634348	0.734777569	0.3616	1.022	0.900	1.161

TGIF1	rs8087619	0.736250035	0.3333	1.022	0.899	1.163
TGIF1	rs387462	0.759908423	0.4046	1.019	0.901	1.153
TGIF1	rs238533	0.76525927	0.1046	1.030	0.850	1.248
TGIF1	rs11661340	0.788672035	0.068	1.032	0.819	1.302
TGIF1	rs16973789	0.800387372	0.04226	1.038	0.775	1.393
TGIF1	rs2238536	0.856415287	0.06429	1.022	0.805	1.300
FBXO11	rs10182633	0.871442037	0.2226	1.012	0.879	1.165
FBXO11	rs13430439	0.888657169	0.1788	1.011	0.868	1.178
TGIF1	rs4797112	0.913086143	0.3602	1.007	0.889	1.141
EVI1	rs12631447	0.926030953	0.3792	1.006	0.895	1.130
TGIF1	rs238135	0.96985284	0.3171	1.002	0.881	1.141
